# Case report: Renal adenoma in a captive ocelot (*Leopardus pardalis*) in Costa Rica

**DOI:** 10.3389/fvets.2024.1393039

**Published:** 2024-05-02

**Authors:** L. Mario Romero-Vega, Sam Medlin, Isabel Hagnauer, Alejandro Alfaro-Alarcón, Bruce Williams

**Affiliations:** ^1^Laboratorio de Patología, Escuela de Medicina Veterinaria, Universidad Nacional, Heredia, Costa Rica; ^2^Joint Pathology Center, Silver Spring, MD, United States; ^3^Rescate Wildlife Rescue Center, Fundación Restauración de la Naturaleza, Alajuela, Costa Rica; ^4^Institute of Virology, Charité–Universitätsmedizin Berlin, Berlin, Germany

**Keywords:** wildlife, neoplasia, renal, ocelot, adenoma

## Abstract

Reports of renal neoplasia are rare in neotropical wildcats. Ocelots (*Leopardus pardalis*) are medium-sized wildcats living in America’s tropical forests. A 12-year-old captive ocelot was diagnosed with a renal mass occupying approximately 25% of the total right kidney volume. The tissue was stained with routine hematoxylin and eosin (H&E) and periodic acid–Schiff (PAS). Immunohistochemistry with the following markers was performed: cytokeratin (CK) AE1/AE3, CK19, CK 7, CD10, vimentin, Melan A, HMB45, Pax-8, and Wilms’ tumor 1 (WT1). Histopathology revealed a well-differentiated epithelial tubular neoplasia with less than one mitotic figure per 2.37mm^2^ field. Vimentin and Pax-8 were the only positive markers. Immunohistochemically, neoplasia was diagnosed as a renal adenoma. Renal adenomas are seldom reported in neotropical wildcats. Reports on wild species are valuable for properly establishing a clinical prognosis for captive species. To the best of our knowledge, this is the first report that provides detailed microscopic and immunohistochemical descriptions of renal adenoma in a captive ocelot.

## 1 Introduction

Wildlife neoplasia is still a developing area in veterinary medicine. Wildlife neoplasia is underreported and rarely detected (1). Most reports are from captive specimens, and the conservation implications of cancer in wildlife are still being discussed and researched. However, it can reduce reproductive success, which alters population dynamics and leads to population declines (2).

The ocelot (*Leopardus pardalis*) is listed as a “Least Concern” species on the International Union for Conservation of Nature (IUCN) red list of threatened species (3). However, in Costa Rica, it is classified as an endangered species (4). Therefore, ocelot populations are threatened by habitat loss, anthropogenic pressure, illegal pet trade, poaching, and logging on a local scale (5, 6).

There are several studies describing neoplasia in captive felids/wildcats. In one study including 40 individuals, the authors did not report any renal neoplasia from nine different felid species. This study did not include species from the *Leopardus* genus (7). Similarly, in a review of 100 feline neoplasias in South Africa, there was no primary kidney neoplasia (8). In another study of 195 non-domestic felids, including 3 ocelots, there was no description of any renal neoplasia (9). Regarding renal adenomas in wild felids, a renal tubular cystadenoma was reported in a lion (*Panthera leo*) (10). In Italy, out of 24 neoplasias from *Panthera* spp., one was a renal adenocarcinoma (11).

Apart from felids, renal adenomas have been described in domestic species, including rats (12), Guinea baboons (13), budgerigars (14), Toco toucans (15), sea horses (16), Red Oscars (17), and several other species (18).

## 2 Case description

**Figure 1:**
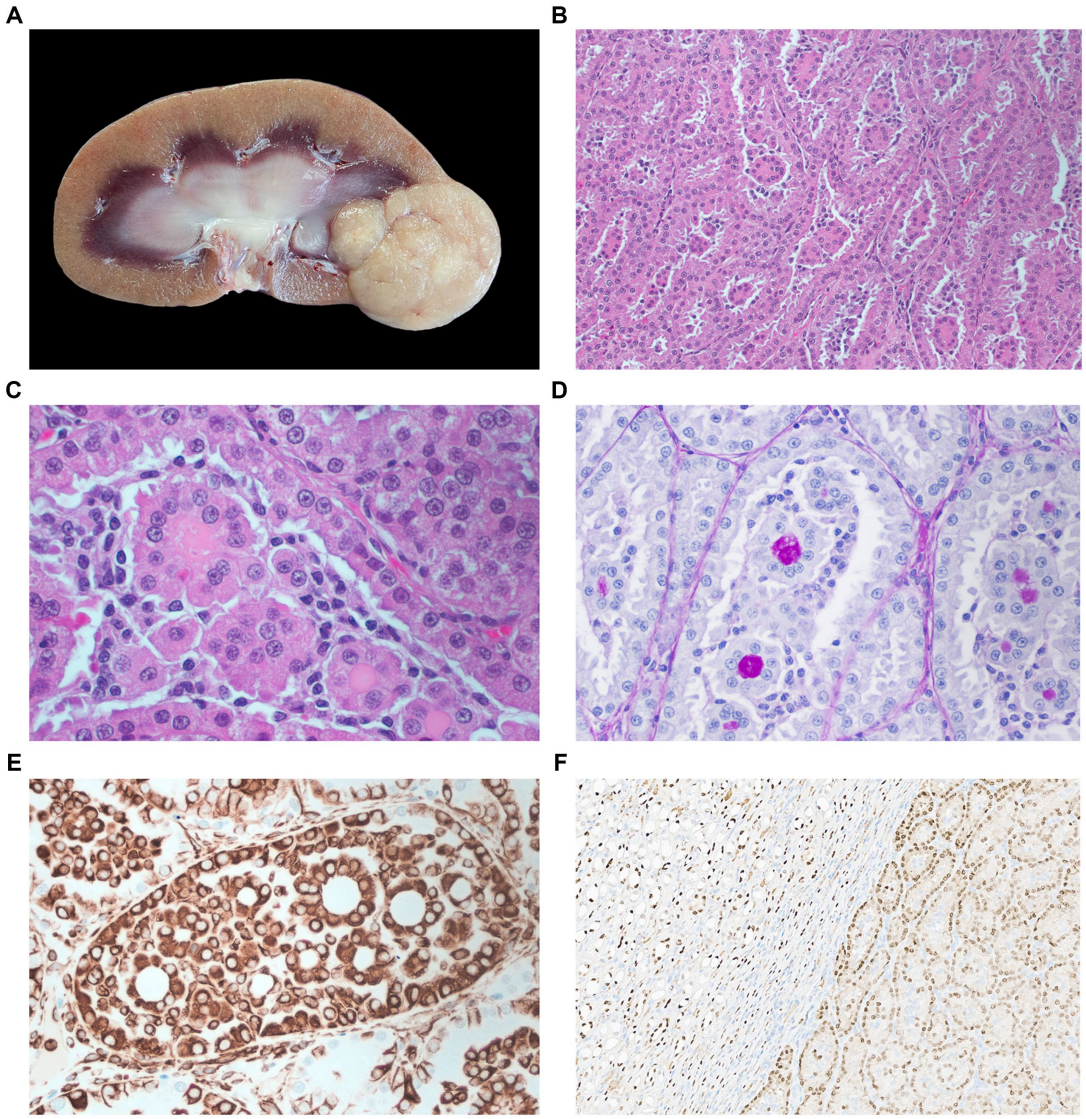
**(A)** Macroscopic appearance of the renal adenoma in the right kidney. The cross-section shows a large beige multilobulated mass occupying one of the renal poles. **(B)** Microphotography of renal adenoma. Tumor tissue grows in a diffuse confluent tubular pattern with the presence of eosinophilic intraluminal proteinaceous material H&E stain 200×. **(C)** Magnification of **B**, scattered tumor-associated lymphocytic infiltration H&E stain 400×. **(D)** Intraluminal PAS-positive material PAS stain 400×. **(E)** Tubular tumor tissue, the majority of the neoplastic cells have moderate to strong cytoplasmic immunoreactivity for vimentin immunohistochemistry staining 400×. **(F)** Strong to moderate immunoreactivity for Pax-8 staining shows the interface between normal renal tissue and neoplasia. Pax-8 immunohistochemistry staining 40×.

A 12-year-old captive male ocelot part of the Rescate Wildlife Rescue Center’s exhibit collection was diagnosed using ultrasound with a renal mass on the cranial pole of the right kidney as part of its annual health check. The differential diagnoses were: renal cyst, lymphoma, carcinoma, adenoma, or an abscess. A decision was made to perform a fine-needle aspiration (FNA) from the mass to obtain a preliminary diagnosis. Cytology showed epithelial cells with moderate anisokaryosis and anisocytosis, naked nuclei, and pseudorosettes consisting of 7–8 cells with a single nucleolus, eosinophilic nucleus, and vacuolated cytoplasm. The preliminary cytological diagnosis was a well-differentiated renal carcinoma. The animal was euthanized 1 week later after the FNA by the veterinary staff using pentobarbital (Euthanex^©^) because of age-related lameness. A full post-mortem examination was performed with no other findings besides a right kidney mass. Focally, a firm, beige, round to oval, and sized 2.6 × 2.2 × 1.6 cm renal mass was observed. This was affecting approximately 25% of the renal tissue. The mass extended from the medulla to the cortex, protruding above the renal surface (Figure 1). After dissection, there was no presence of hemorrhage or necrosis on the cutting surface. The kidney was placed in 10% buffered formalin. After trimming, the sample was processed for routine hematoxylin and eosin (H&E) and periodic acid–Schiff (PAS) staining. Several immunohistochemistry (IHC) markers were chosen to characterize the likely cellular origin of the neoplasia (Table 1).

**Table 1:** Immunohistochemistry positivity with antibody host, source, antigen retrieval method, and chromogen.

Target antigen	Positivity	Host	Source	Antigen retrieval	Chromogen
CK AE1/AE3	−	Mouse	Leica Biosystems PA 0909	Proteinase K	DAB
CK19	−				DAB
CK 7	−	Mouse	Leica Biosystems 0942	ER1	DAB
CD10	−				
Vimentin	+	Mouse	Ventana Medical Systems 790-2917	ER1	DAB
Melan-A	−	Mouse	Leica Biosystems PA 0223	ER2	DAB
HMB-45	−	Mouse	Ventana Medical Systems 790-4366	Proteinase K	DAB
Pax-8	+	Mouse	Leica Biosystems MRQ-50	ER2	DAB
WT1	−	Mouse	Leica Biosystems WT49	ER2	DAB

Microscopically (Figure 1), the neoplasm was well-demarcated, densely cellular, and non-encapsulated with expansile growth. Neoplastic cells formed a diffuse tubular pattern without significant tubule-papillary or papillary formation. The neoplastic cells are supported by a fine fibrovascular stroma. Cytological and nuclear features are well-differentiated cubical epithelial cells with abundant eosinophilic cytoplasm. The nuclei are centered, with scattered chromatin and a single nucleolus, with minimal signs of nuclear atypia. The mitotic count was less than 1 per 2.37mm^2^ field. There was no infiltrative growth or metastasis. The renal tissue surrounding the neoplastic cells is moderately compressed, without evidence of necrosis. There was also scattered tumor-associated lymphocytic infiltration.

The PAS stained a highly positive material contained in the lumen of the acinar structures of the adenoma. Based on published markers for veterinary and human renal tumors (19–21), we selected eight antibodies for immunohistochemical characterization (Table 1). It was found that tumor tissue was only positive for two IHC markers. Epithelial cells forming the tubules and acinar structures had a positive cytoplasmic labeling for vimentin and a positive nuclear labeling for Pax-8 immunomarkers (Figure 1).

## 3 Discussion

Well-differentiated renal carcinomas histologically make it challenging to differentiate them from renal adenomas (17). Therefore, a correct diagnosis is critical to establishing a clinical prognosis. To the best of our knowledge, this is the first renal adenoma reported in the *Leopardus* genus, which includes 13 New World felids. Adenomas are scarcely reported in other wild cats; for instance, in one study of 108 large felid neoplasias, one tiger (*Panthera tigris*) had a renal adenoma (22). Furthermore, a jungle cat (*Felis chaus*) was reported to have multicentric adenomas, including thyroid, gastric, and renal adenomas (23), and a puma with a renal cortical adenoma (24).

The occurrence in an ocelot is valuable since this species’ neoplasia is rarely reported in Latin America. Recently, other neoplasias have been documented in ocelots, including a seminoma, pulmonary adenocarcinoma (25), and transitional cell carcinoma (26).

Adenomas are rare tumors in domestic animals and are usually clinically silent (27). Nonetheless, depending on the anatomical position within the renal parenchyma, they can obstruct the urinary flow and develop hydronephrosis (28). Furthermore, it has been reported that hypertrophic osteopathy can occur concomitantly with renal adenomas in cats (29). In other lesions and anatomical locations, adenomas are well-known to be premalignant lesions; for example, colorectal adenoma is a precancerous lesion for colorectal carcinoma (30). For renal carcinomas, there is evidence in humans that papillary adenomas and renal cell carcinoma may arise from the same precursor lesion based on chromosomal mutations (31). In veterinary medicine, this progression from adenoma to carcinoma has been chiefly explored for mammary neoplasia (32, 33).

This renal carcinoma case was well-differentiated and exhibited histologic features similar to those described in adenomas in dogs and cats. In domestic small animal species, an arbitrary cutoff of 2 cm or larger is used to differentiate renal carcinoma from benign lesions. Nonetheless, this size cutoff for adenoma and carcinoma in exotic/wildlife species has yet to be established. Other criteria for differentiating renal carcinoma from benign lesions include the presence of metastases, increased mitotic count, and invasion into surrounding tissues, as well as the presence of anaplastic cellular and nuclear features (27). All these malignant criteria were absent in this case. The only malignant criterion observed in this case, according to domestic classification standards, was the size of neoplasia, which measured 2.6 cm (greater than 2 cm). Based on these findings, the diagnosis of this neoplasia as an adenoma over a well-differentiated carcinoma. The positive PAS material in the lumen of the acinar structures is most likely glycoproteins, which may contribute to the formation of urinary casts (Figure 1). Although there are several PAS-positive urinary casts, the ones composed of Tamm–Horsfall protein (uromodulin) have a very strong PAS staining (34) similar to this case. This protein is secreted by the epithelium of the thick ascending limb of the loop of Henle and early distal tubules (35).

Vimentin and Pax-8 were the only positive markers for the IHC. Vimentin is an intermediate filament used as a positive marker for mesenchymal tumors, but it can label several carcinomas (36). In this case, vimentin moderately labeled normal collecting tubules and distal tubules. In domestic cats, vimentin has been reported to be expressed in distal tubules and collecting ducts but not in proximal tubules (19). Vimentin can positively stain several subtypes of renal carcinomas originating from different renal tubules (22, 36, 37). In humans, this generalized positive labeling of vimentin in renal carcinomas is attributed to an epithelial-mesenchymal transition (38); nonetheless, this hypothesis has not been thoroughly investigated in veterinary medicine for renal carcinomas. In this same study, cytokeratin (CK) AE1/AE3 labeled normal distal tubules, collecting ducts, and renal pelvic epithelium, while CK7 only labeled collecting ducts and renal pelvic epithelium. Although CK AE1/AE3 is commonly used as an epithelial marker, in this case, it did not label the neoplastic cells. This could suggest that this adenoma could have proliferated from proximal tubules since they are not labeled by this marker. In humans, it has been reported that metanephric adenoma is negative for CK7 staining (39).

Pax-8 is a nuclear marker for epithelial cells in several tissues, including renal, thyroid, and endometrial (40). It has been established in human and veterinary medicine as a marker to help detect different neoplasias of renal origin, including renal carcinoma (20). In dogs, it has been established as a positive marker for renal thyroid and upper Müllerian tract neoplasms (41). To the best of our knowledge, there are no other reports of the use of Pax-8 for renal adenomas in veterinary medicine. In human medicine, it has been reported as a positive marker for nephrogenic adenoma (42).

We also performed labeling for HMB45 and Melan-1 since tissue positivity has been reported in renal carcinoma in humans (21). Finally, there are no specific markers for nephroblastoma (Wilms’ tumor) in veterinary medicine. Nonetheless, we used it as it has been done in other veterinary cases (43).

Regarding the specific cellular origin of this renal adenoma, there is no clear evidence of the origin. The vimentin-positive marking and the presence of presumptive Tamm–Horsfall protein suggest that it could have a distal tubule origin. Nonetheless, the negative CK AE1/AE3 suggests that it could have a proximal tubule origin.

Wildlife neoplasia and exotic species reports are still infrequent. New reports such as this case contribute to the further characterization of this pathological process. Unfortunately, the availability of species-specific IHC markers and the small number of reports limit the development of proper tumor classification guidelines. From a clinical point of view, reports and immunophenotyping of these adenomas can help in establishing the origin of neoplasia in cases of metastasis with an established tissue origin. This information is useful for the prognosis and clinical management of this species, contributing to the conservation of the species, especially in individuals involved in an *ex situ* reproduction program aimed at enhancing genetic diversity in free-ranging populations.

## Data availability statement

The original contributions presented in the study are included in the article/supplementary material, further inquiries can be directed to the corresponding author.

## Ethics statement

Written informed consent was obtained from the owners for the participation of their animals in this study.

## Author contributions

LR-V: Conceptualization, Data curation, Methodology, Writing – original draft, Writing – review & editing. SM: Formal analysis, Writing – original draft. IH: Methodology, Writing – original draft. AA-A: Conceptualization, Funding acquisition, Writing – review & editing. BW: Supervision, Writing – review & editing.
